# Novel Recombinant Multiepitope Proteins for the Diagnosis of Asymptomatic *Leishmania infantum*-Infected Dogs

**DOI:** 10.1371/journal.pntd.0003429

**Published:** 2015-01-08

**Authors:** Angélica Rosa Faria, Luciano de Castro Veloso, Wendel Coura-Vital, Alexandre Barbosa Reis, Leonardo Miranda Damasceno, Ricardo T. Gazzinelli, Hélida M. Andrade

**Affiliations:** 1 Universidade Federal de Minas Gerais, Instituto de Ciências Biológicas, Departamento de Parasitologia, Belo Horizonte, Minas Gerais, Brasil; 2 Universidade Federal de Ouro Preto, Núcleo de Pesquisa em Ciências Biológicas, Ouro Preto, Minas Gerais, Brasil; 3 Universidade Federal de Minas Gerais, Instituto de Ciências Biológicas, Departamento de Bioquímica e Imunologia, Belo Horizonte, Minas Gerais, Brasil; 4 Centro de Pesquisas René Rachou – Fundação Oswaldo Cruz, Belo Horizonte, Minas Gerais, Brasil; 5 University of Massachusetts Medical School, Division of Infectious Diseases and Immunology, Worcester, Massachusetts, United States of America; Barcelona Centre for International Health Research (CRESIB), Spain

## Abstract

**Background:**

Visceral leishmaniasis is the most severe form of leishmaniasis. Worldwide, approximately 20% of zoonotic human visceral leishmaniasis is caused by Leishmania infantum, also known as Leishmania chagasi in Latin America. Current diagnostic methods are not accurate enough to identify Leishmania-infected animals and may compromise the effectiveness of disease control. Therefore, we aimed to produce and test two recombinant multiepitope proteins as a means to improve and increase accuracy in the diagnosis of canine visceral leishmaniasis (CVL).

**Methodology/Principal Findings:**

Ten antigenic peptides were identified by CVL ELISA in previous work. In the current proposal, the coding sequences of these ten peptides were assembled into a synthetic gene. Furthermore, other twenty peptides were selected from work by our group where good B and T cell epitopes were mapped. The coding sequences of these peptides were also assembled into a synthetic gene. Both genes have been cloned and expressed in Escherichia coli, producing two multiepitope recombinant proteins, PQ10 and PQ20. These antigens have been used in CVL ELISA and were able to identify asymptomatic dogs (80%) more effectively than EIE-LVC kit, produced by Bio-Manguinhos (0%) and DPP kit (10%). Moreover, our recombinant proteins presented an early detection (before PCR) of infected dogs, with positivities ranging from 23% to 65%, depending on the phase of infection in which sera were acquired.

**Conclusions/Significance:**

Our study shows that ELISA using the multiepitope proteins PQ10 and PQ20 has great potential in early CVL diagnosis. The use of these proteins in other methodologies, such as immunochromatographic tests, could be beneficial mainly for the detection of asymptomatic dogs.

## Author Summary

Visceral leishmaniasis is the most severe form among leishmaniasis, being a neglected disease caused by a protozoan parasite. Its transmission through phlebotominae bites, between dogs and humans, classifies it as a zoonotic disease. It is caused by the specie *Leishmania infantum* ( = *L. chagasi*) and represents 20% of the world's human visceral leishmaniasis. Visceral leishmaniasis is a serious public health issue, fatal if untreated, and its incidence is increasing in urban areas of the tropics. In Brazil, one of the control measures is the identification and elimination of infected dogs, which act as reservoirs for *Leishmania* parasites. Diagnostic methods used to identify infection in these animals are still not accurate enough, which may compromise the effectiveness of this control measure. Thus, to contribute to the diagnosis of canine visceral leishmaniasis, we aimed to develop and test two new antigens that could be applied in early detection of infected dogs.

## Introduction

Visceral leishmaniasis is caused by a protozoan parasite and affects approximately 500,000 million individuals annually worldwide [1]. Dogs are the main domestic reservoir of the causative agent of zoonotic visceral leishmaniasis, *Leishmania infantum* ( = *L. chagasi*) [2], [3].

Measures employed to control visceral leishmaniasis in Brazil since the 80's involve the elimination of infected dogs, among other actions [4]. Many serological tests have been described to detect canine anti-leishmanial antibodies [5]–[7]. However, current diagnostic tests lack sufficient sensitivity or specificity, including those recommended by the Brazilian Ministry of Health, Dual-Path Platform (DPP; Bio-Manguinhos/Fiocruz, Rio de Janeiro, Brazil) and Canine Leishmaniasis ELISA kit (EIE-LVC kit; Bio-Manguinhos/Fiocruz, Rio de Janeiro, Brazil) [8],[9]. Thus, canine visceral leishmaniasis (CVL) represents a serious public health issue given the frequency of asymptomatic infections (up to 85%) [10], and given that both asymptomatic and symptomatic dogs are equally infectious to the vectors [11], [12]. Therefore, the development of accurate diagnostic methods for canine infection, mainly for asymptomatic animals, is essential for visceral leishmaniasis surveillance programs, in addition to understanding immunological responses in resistant or susceptible animals.

Recently, an increasing number of *Leishmania* antigens have been evaluated in serodiagnosis [8], [13]–[16]. High values of sensitivity and specificity are very important to these antigens. However, if the objective is a screening test, high sensitivity is desirable. But if a confirmatory test is being developed, high specificity becomes more important in this case. In an attempt to achieve high sensitivities and specificities in tests, an alternative approach is the use of multiepitope proteins, which have been demonstrated to be a valuable tool in CVL diagnosis. Soto et al. [17] evaluated a chimeric protein for the diagnosis of *L. infantum*-infected (n = 59) and uninfected dogs (n = 15) and showed 79% of sensitivity and 96% of specificity. Boarino et al. [18] tested another chimeric antigen in 232 animals in which leishmanial infection had been detected by parasitological examination (n = 19) or RIFI (n = 213). They showed that this chimeric antigen had 96% of sensitivity and 99% of specificity in this diagnosis. Despite these promising results, these antigens were tested only in the Old World. Therefore, tests with other multiepitope proteins are still needed, not only in other geographical areas, but also in dogs with different clinical status, in comparison to molecular assays and in follow up of canine infection.

Early serological detection of CVL is highly desirable, mainly because in urban areas there is a high prevalence of infected dogs (as determined by PCR) that are not detected by conventional serological methods [19]. Therefore, we aimed to search for new antigens that could be used to detect CVL in an early phase.

In this study, we constructed two multiepitope proteins using epitopes previously identified [20]. The protein named PQ20, constituted by twenty peptides, presented approximately 95% of B cell epitopes when mapped with BCPreds and ABCPred programs. The other protein, named PQ10, was constituted by ten antigenic peptides that showed good results in CVL ELISA, with accuracy up to 0.94. Some of them gave positive reactions in up to 95% of asymptomatic dog sera. When these peptides were mixed into a single solution, good results were also obtained [8]. These two multiepitope proteins were tested in asymptomatic dog detection and also in early detection (before PCR positive results) of CVL. In this work, the main contribution of multiepitope antigens would be the usefulness in canine management, due to their ability to detected infected animals in a serological test, with similar sensitivitiy to PCR.

## Methods

### Canine sera

Serological tests were performed in 3 steps: 1) First, we used a sera panel (n = 52) in ELISA with the multiepitope proteins, tested in parallel with DPP and EIE-LVC kit; 2) To validate ELISA with the multiepitope proteins, we used a multicentric panel with 131 serum samples (negative in indirect immunofluorescence reaction - RIFI and EIE-LVC kit) and 231 serum samples that were positive in RIFI and EIE-LVC kit; 3) A panel of 42 serum samples was tested in ELISA with the multiepitope proteins; these sera were acquired from dogs which were followed for 18 months and were initially negative in PCR and EIE-LVC kit.

In the first step, the uninfected dogs (n = 9) were negative based on parasitological and serological tests (RIFI and EIE-LVC kit), while infected animals (n = 43) were certified by parasitological tests conducted on bone marrow cells examined by optical microscopy, and also by RIFI and EIE-LVC kit. Within samples of infected dogs, n = 33 were clinically undefined while n = 10 were asymptomatic according to typical CVL signs, stated elsewhere [21].

In the second step, ELISA validation, we used a multicentric panel with 362 samples, of which n = 131 negative in RIFI and EIE-LVC kit. Within this negative group, n = 40 serum samples were from animals born in kennels with wire mesh in a non-endemic area (Ouro Preto, Minas Gerais, Brazil; Southeast), being the N1 subgroup. Then, n = 91 serum samples were from endemic area animals (Belo Horizonte, Minas Gerais, Brazil; Southeast). Within these samples, n = 22 had negative results by PCR, being the N2 subgroup; the others (n = 69), which were not tested by PCR, were classified as N3 subgroup. Conversely, n = 231 serum samples had positive results in RIFI and EIE-LVC kit, and were clinically undefined. Within this group, n = 87 samples were from endemic area animals (Teresina, Piauí, Brazil; Northeast) and were also positive in the DPP test, being the P1 subgroup. The other samples (n = 144) were from dogs living in another endemic area (Belo Horizonte, Minas Gerais, Brazil). Among them, n = 22 samples were also positive by PCR, being the P2 subgroup; the other n = 122 samples were not tested by PCR, being the P3 subgroup (Table 1).

**Table 1:** Canine serum samples used in validation of ELISA-PQs.

Subgroups	Tests				Number of samples	Additional information
	EIE-LVC kit	RIFI	DPP	PCR		
**N1**	-	-	NP	NP	n = 40	Animals born in kennels from non-endemic area*
**N2**	-	-	NP	-	n = 22	Animals from endemic area#
**N3**	-	-	NP	NP	n = 69	Animals from endemic area#
**P1**	+	+	+	NP	n = 87	Animals from endemic area£
**P2**	+	+	NP	+	n = 22	Animals from endemic area#
**P3**	+	+	NP	NP	n = 122	Animals from endemic area#

NP: not performed;

+: positive result;

-: negative result;

*: Ouro Preto, Minas Gerais state, Brazil;

# : Belo Horizonte, Minas Gerais state, Brazil;

£ : Teresina, Piauí state, Brazil.

Finally (third step), samples from a greater cohort study [22] were included in this work. Canine blood was collected in three different periods: in the beginning of the cohort (T0), 12 months later, and 18 months later. During all this time, they were maintained in their households, exposed to all the risk factors. Sera from these dogs (n = 42) were tested by PCR-RFLP, EIE-LVC kit, as previously described [22], and ELISA with multiepitope proteins (PQ10 and PQ20). All animals were from Belo Horizonte, Minas Gerais state, Brazil, and were followed to verify seroconversion in this endemic area.

All serum samples came from already existing collections, from Laboratório de Leishmanioses of Universidade Federal de Minas Gerais, Minas Gerais state, Brazil and from Laboratório de Imunoparasitologia of Universidade Federal de Ouro Preto, Minas Gerais state, Brazil.

### Design of synthetic genes and production of recombinant proteins

Two different multiepitope synthetic genes were designed. First, we joined 10 coding sequences from antigenic peptides [8], resulting in PQ10. Second, we joined 20 coding sequences from T and B cell epitopes [20], resulting in PQ20. A flexible linker (Gly-Ser-Gly-Ser-Gly) coding sequence was used as a spacer between epitope sequences [23]. *Nde*I and *Not*I restriction sites were added to the 5′ and 3′ ends, respectively, of both synthetic genes to aid in cloning. A 6xHIS tag coding sequence was added upstream of the stop codon of each synthetic gene for affinity purification of recombinant proteins. Both sequences were codon-optimized for *Escherichia coli* expression. PQ10 and PQ20 genes were commercially synthesized by Genscript, USA.

Synthetic genes were cloned into the *Nde*I and *Not*I restriction sites of a pET9a24a expression vector, resulting in pET-PQ10 and pET-PQ20. Sequence analysis of the cloned fragments confirmed the correct fusion and orientation of the insert. Recombinant plasmids were used to transform *E. coli* C41 strain and protein expression was carried out by inoculating 500 mL of Luria Bertani medium containing 0.05 mg/mL kanamycin with an overnight bacterial culture. This suspension was incubated on a rotary shaker (200 rpm) at 37°C until an optical density of 0.6 at 600 nm. Protein expression was induced with 0.4 mM IPTG (isopropyl-β-D-thiogalactopyranoside) for 4 h on a rotary shaker (200 rpm) at 37°C. Cells were lysed using a microfluidizer (EmulsiFlex C3, Avestin) and soluble and insoluble protein fractions were analyzed by SDS-PAGE [24]. Next, insoluble fractions of recombinant proteins were affinity purified using an ÄKTA Prime chromatography system (GE Healthcare Life Science) with a 5 mL HIS-Trap FF column (GE Healthcare Life Science), in the presence of 8 M urea, according to manufacturer's instructions.

### Immunoassays with canine sera

To evaluate the antigenicity of multiepitope proteins, ELISA was conducted with both PQ10 and PQ20. All ELISA procedures were optimized in terms of antigen concentrations, dilutions of serum and conjugated immunoglobulins, and the microplates that would be employed. Falcon flexible microplates (Becton Dickinson®, France) for PQ10 and Eppendorf microplates (Hamburg, Germany) for PQ20 were coated for 16 h approximately with 0.5 µg/mL recombinant proteins diluted in 0.05 M carbonate buffer (pH 9.6) at 4°C. After three washes with PBS/T (PBS: 10.14 mM Na2HPO4; 1.37 mM KH2PO4; 146 mM NaCl; 2.64 mM KCl, pH 7.4, containing 0.05% Tween20), wells were blocked with 5% powdered skimmed milk in PBS/T at 37°C for 1 h. Serum samples, diluted 1∶200 in PBS/T containing 0.5% powdered skimmed milk, were added and incubated at 37°C for 1 h. After three washes, plates were incubated with peroxidase-conjugated anti-dog immunoglobulin G (Sigma, reference A9042), diluted 1∶2500 in PBS/T containing 0.5% powdered skimmed milk at 37°C for 1 h. After washing three times, reactions were developed with Fast-OPD (Sigma) and plates were incubated for 30 min in the dark, following manufacturer's instructions. Reactions were stopped with 2 M H_2_SO_4_, and plates were read at 492 nm in a MultiskanGo (ThermoScientific) plate reader.

Samples that provided discordant results (positive in RIFI and EIE-LVC kit but negative in ELISA with PQ10 and PQ20) were tested by another methodology. The immunochromatographic test Kalazar Detect (InBios) was used according to manufacturer's instructions. Additionally, anti-CVL antibody concentration in serum samples was titrated by RIFI [25].

### Statistical analysis

Recombinant proteins sensitivity and specificity were calculated using parasitological results as a gold standard. A cut off point for optimal sensitivity and specificity was determined using ROC analysis [26], and the area under the curve (AUC) was calculated to assess performance of the tests. Once the intended use of multiepitope proteins is in both screening and confirmatory tests, the highest values of sensitivity and specificity were desirable. All of the statistical analyses were performed using GraphPad Prism (version 5.0).

## Results

### Multiepitope protein production

**Figure 1:**
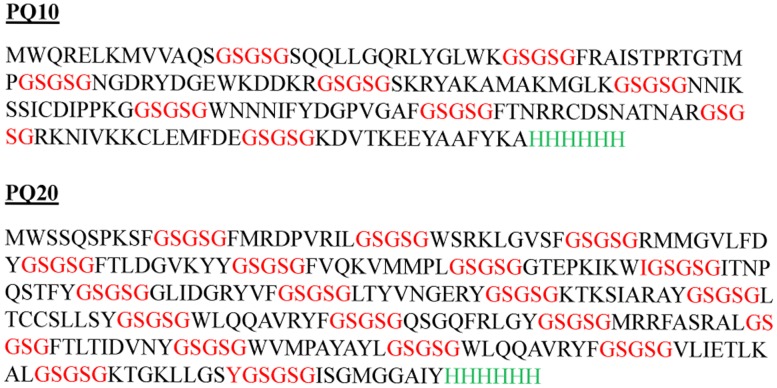
PQ10 and PQ20 amino acid sequences. Predicted amino acid sequences of recombinant proteins PQ10 and PQ20, showing flexible linkers in red and 6XHIS tag in green.

**Figure 2:**
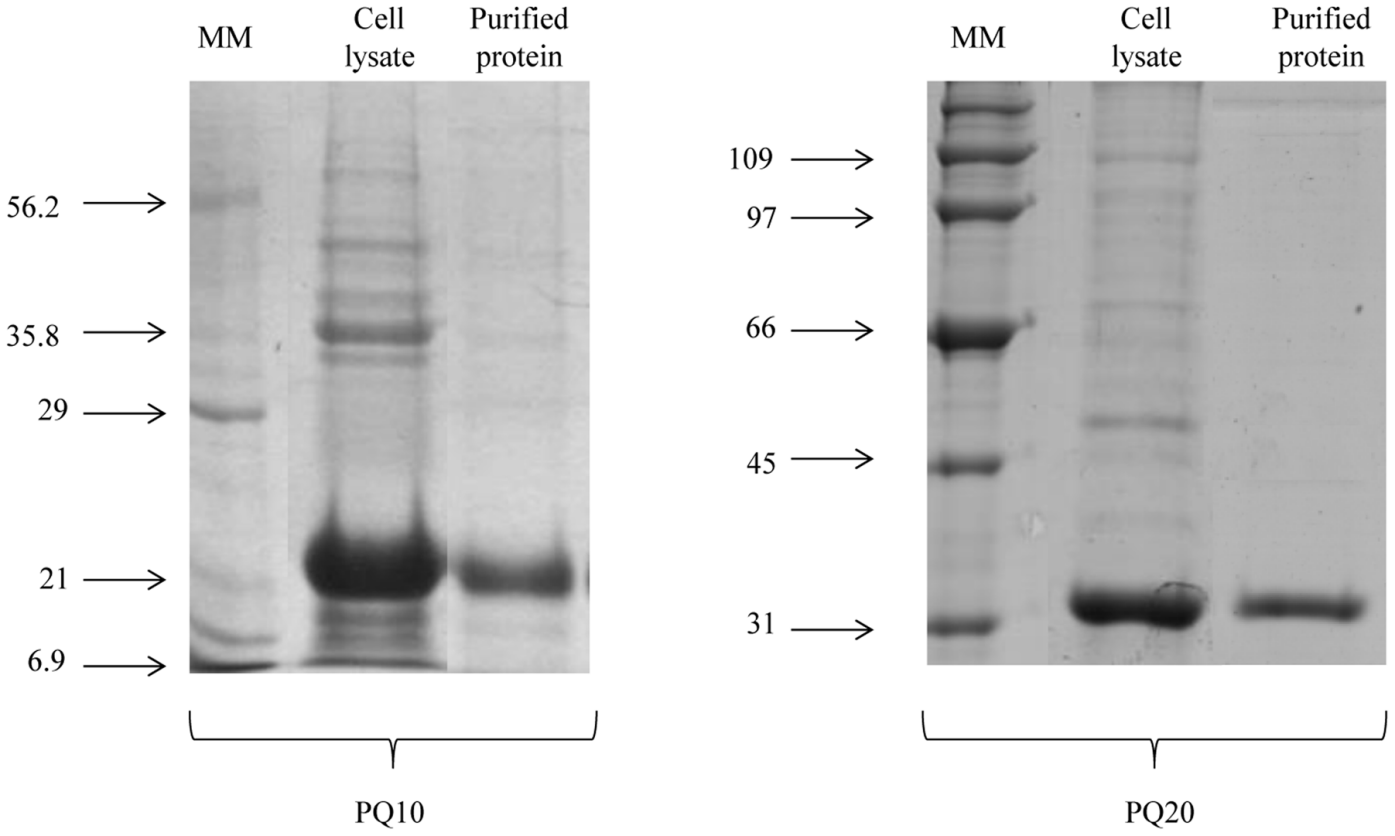
Cell lysate fractions and purified multiepitope proteins. Coomassie blue-stained 12% SDS-PAGE analysis of cell lysate insoluble fraction and purified fractions resulting from IPTG-induced bacterial cultures, showing bands in the expected sizes for the recombinant proteins. MM: molecular mass in kDa.

The two multiepitope proteins, PQ10 and PQ20, were successfully produced by *E. coli* C41 strain under the conditions described in Methods. Amino acid sequences (with flexible linkers) are shown in Fig. 1. Fig. 2 shows SDS-PAGE analysis of highly expressed recombinant proteins at the expected sizes. A band of approximately 21 kDa, referring to PQ10, and another of approximately 33 kDa, referring to PQ20, were observed. Both recombinant proteins were found mainly in the insoluble fraction of the cell lysate. Fig. 2 also shows fractions of the purified multiepitope proteins.

### Comparison between tests – asymptomatic detection

When comparing the four different CVL diagnostic tests: 1) DPP; 2) EIE-LVC kit; 3) ELISA with PQ10; and 4) ELISA with PQ20, large differences were found. These tests were performed with sera from uninfected (n = 9) and infected dogs (n = 43), as described in Methods. While sensitivities of EIE-LVC kit and DPP were 64.5% and 72.9% respectively, ELISA-PQ10 and ELISA-PQ20 showed sensitivities of 88.8% and 84.9%, respectively (Table 2). However, ELISA-PQ10 and ELISA-PQ20 (ELISA-PQs) specificities were lower (PQ-10: 80% and PQ-20: 65%) when compared to other test specificities (DPP: 90% and EIE-LVC kit: 100%). Additionally, multiepitope protein PQ10 was able to detect 80% of asymptomatic infected dogs, which was not observed with EIE-LVC kit. This test showed no asymptomatic dog detection while DPP detected only 10% of them.

**Table 2:** Comparative performance of CVL diagnostic tests.

	DPP	EIE-LVC kit	ELISA-PQ10	ELISA-PQ20
**Sensitivity (%)**	72.9	64.5	88.8	84.9
**Specificity (%)**	90	100	80	65

### Validation of ELISA-PQs

**Figure 3:**
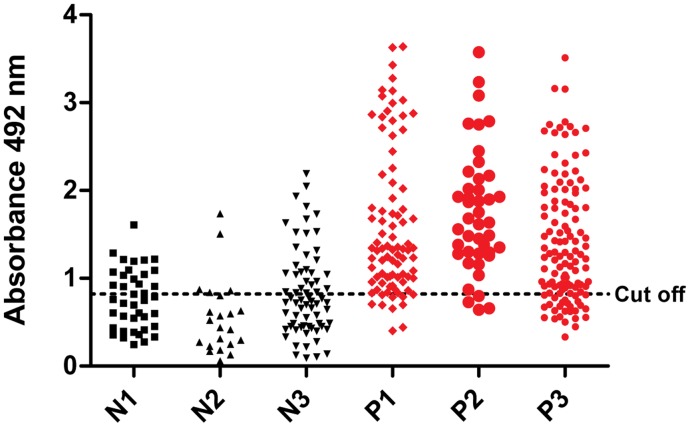
Comparison of ELISA reactivity of canine sera against PQ10. ELISA was performed in different groups of dogs (N: negative group; P: positive group). Subgroup N1: samples with negative results in EIE-LVC kit and RIFI, from animals born in kennels with wire mesh in a non-endemic area (Ouro Preto, Minas Gerais state, Brazil). From an endemic area (Belo Horizonte, Minas Gerais state, Brazil), subgroup N2 shows samples with negative results in EIE-LVC kit, RIFI and PCR; subgroup N3 shows samples with negative results in EIE-LVC kit and RIFI. Subgroup P1: samples with positive results in EIE-LVC kit, RIFI and DPP, from endemic area animals (Teresina, Piauí state, Brazil). From other endemic area (Belo Horizonte, Minas Gerais state, Brazil), subgroup P2 shows samples with positive results in EIE-LVC kit, RIFI and PCR; subgroup P3 shows samples with positive results in EIE-LVC kit and RIFI.

**Figure 4:**
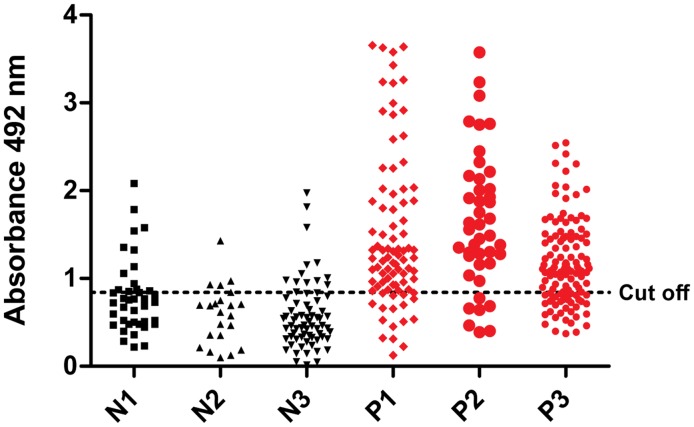
Comparison of ELISA reactivity of canine sera against PQ20. ELISA was performed in different groups of dogs (N: negative group; P: positive group) Subgroup N1: samples with negative results in EIE-LVC kit and RIFI, from animals born in kennels with wire mesh in a non-endemic area (Ouro Preto, Minas Gerais state, Brazil). From an endemic area (Belo Horizonte, Minas Gerais state, Brazil), subgroup N2 shows samples with negative results in EIE-LVC kit, RIFI and PCR; subgroup N3 shows samples with negative results in EIE-LVC kit and RIFI. Subgroup P1: samples with positive results in EIE-LVC kit, RIFI and DPP, from endemic area animals (Teresina, Piauí state, Brazil). From other endemic area (Belo Horizonte, Minas Gerais state, Brazil), subgroup P2 shows samples with positive results in EIE-LVC kit, RIFI and PCR; subgroup P3 shows samples with positive results in EIE-LVC kit and RIFI.

Validation was performed with 231 serum samples from *L. infantum*-infected dogs and 131 serum samples from uninfected dogs as the negative controls. Promising results were achieved with ELISA-PQs, which provided global sensitivities of 80.2% (PQ10) and 84.9% (PQ20). The specificity was the same for both, 65.6%. According to Swets [27], these antigens provided tests with moderate accuracy (AUC = 0.82 for PQ10 and AUC = 0.84 for PQ20). In Fig. 3 and 4, the absorbance distribution of ELISA-PQ10 and ELISA-PQ20, respectively, is shown, as well as the cut off point for optimal sensitivity and specificity. Analyzing the different subgroups, we observed that between those with only two serological concordant tests (RIFI and EIE-LVC kit), N3 and P3, specificities are lower when compared to those groups, N2 and P2, with an additionally parasitological concordant test, as PCR (Table 3). Specificities ranged from 65.5% to 71.0% when comparing subgroups N3 and P3 and from 90.9 to 72.7 in the other comparison (N2 and P2). We observed that a third methodology to guarantee CVL diagnosis (born in kennels with wire mesh or an additional concordant test such as DPP or PCR) decreases the number of false diagnosis by PQs. Furthermore, it was possible to notice that ELISA-PQs' performance were similar when testing samples from different places. Samples from groups P1 (from Teresina, Piauí state, Brazil; Northeast) and P3 (from Belo Horizonte, Minas Gerais state, Brazil; Southeast) for example, showed similar results, indicating that the antigens present satisfactory performance in different CVL endemic areas. Moreover, besides the fact that ELISA-PQs presented greater sensitivities and specificities in groups with concordant parasitological and serological results, these results also indicate that these novel antigens could lead to improvements of diagnostic tests.

**Table 3:** Diagnostic performance of ELISA-PQs in different groups of dogs.

Groups	Sensitivity (%)		Specificity (%)		AUC	
	PQ10	PQ20	PQ10	PQ20	PQ10	PQ20
**N1 (40)/P1 (87)**	80.4	80.4	65.0	72.5	0.84	0.78
**N2 (22)/P2 (22)**	90.7	86.0	90.9	72.7	0.93	0.89
**N3 (69)/P3 (122)**	74.5	88.5	65.5	71.0	0.75	0.86

N: negative group. Subgroup N1: samples with negative results in EIE-LVC kit and RIFI, from animals born in kennels with wire mesh in a non-endemic area (Ouro Preto, Minas Gerais state, Brazil). From an endemic area (Belo Horizonte, Minas Gerais state, Brazil), subgroup N2 shows samples with negative results in EIE-LVC kit, RIFI and PCR; subgroup N3 shows samples with negative results in EIE-LVC kit and RIFI. P: Positive group. Subgroup P1: samples with positive results in EIE-LVC kit, RIFI and DPP, from endemic area animals (Teresina, Piauí state, Brazil). From other endemic area (Belo Horizonte, Minas Gerais state, Brazil), subgroup P2 shows samples with positive results in EIE-LVC kit, RIFI and PCR; subgroup P3 shows samples with positive results in EIE-LVC kit and RIFI.

To investigate the occurrence of false-negative results in ELISA-PQs, samples that showed discordant results were tested by Kalazar Detect. This method provided only 56% of positivity when testing samples that were negative in ELISA-PQs and positive in EIE-LVC and RIFI. Thus, to test if these samples had low titers of anti-*Leishmania* antibodies, a quantitative RIFI was performed, until a maximum titer of 1∶2560. Indeed, the majority of samples had low titers of antibodies, with only 9.5% of them (from n = 63) possessing titers above 1∶320. It showed that ELISA-PQs cannot easily react with serum samples that have low antibody titers.

### Detection of *L. infantum* infection in early phase

**Figure 5:**
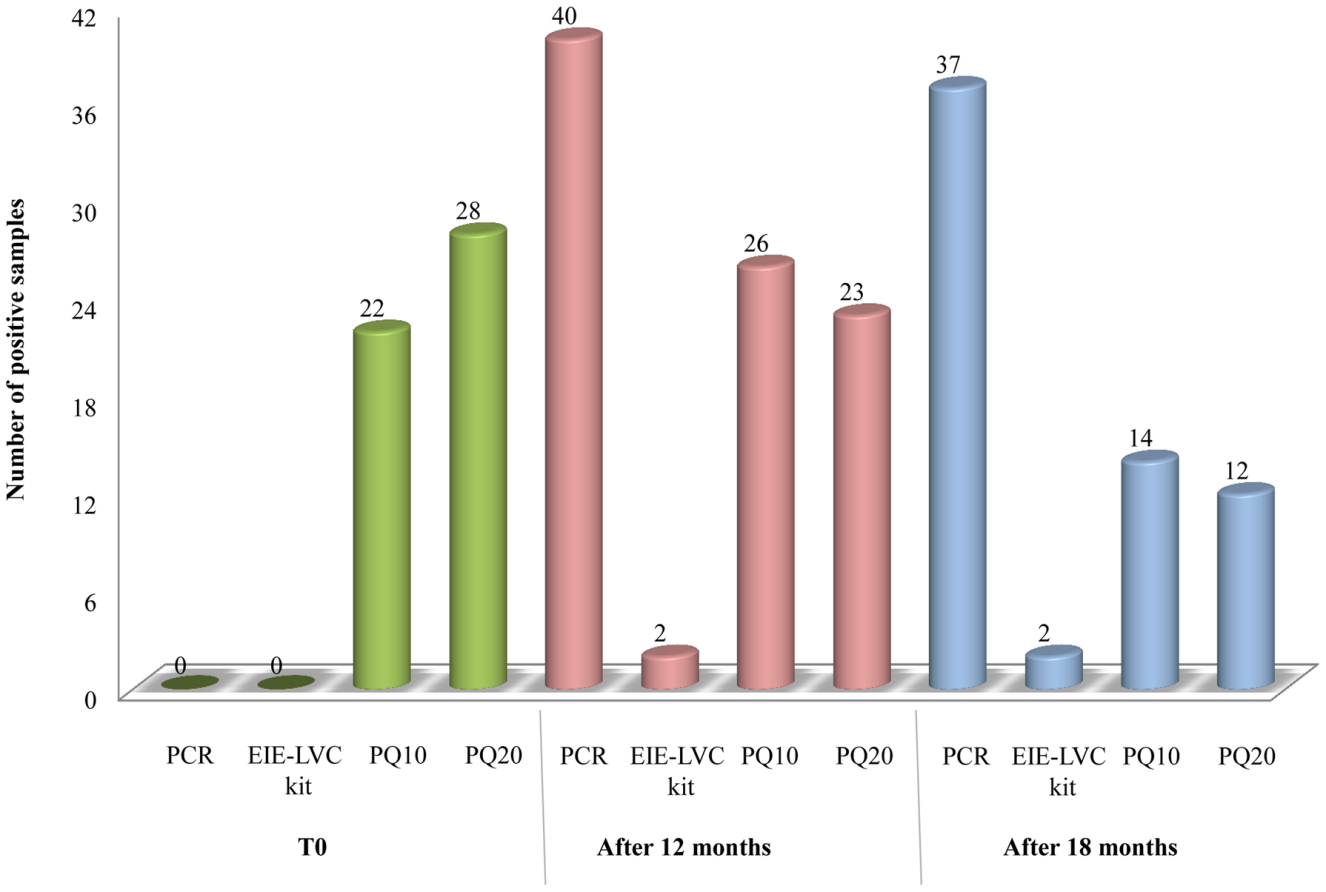
Positivities of canine sera against PCR, EIE-LVC kit and ELISA-PQs. Canine sera was obtained in a first analysis (T0), after 12 months and after 18 months, and tested against PCR, EIE-LVC kit and ELISA-PQs.

Samples from n = 42 CVL endemic area dogs (negative in PCR and EIE-LVC kit) were analyzed by ELISA-PQs and were followed for 18 months. In the first analysis (T0), ELISA-PQ10 already identified 51.1% of the samples as positive and ELISA-PQ20, 65.1% (Fig. 5). Afterwards, PCR provided positive results, reaching a positivity of 86.0% after 18 months. These positivities were not accompanied by EIE-LVC kit, which detected only 4.6% of the cases starting at the second analysis (after 12 months). Recombinant proteins were able to detect positive dogs starting at the first analysis, detecting a similar number of cases after 12 months, with a decrease after 18 months. This suggests that the antibodies detected by ELISA-PQs reach maximum levels just after infection, decreasing thereafter. Besides, all animals which provided ELISA PQs' positive results in T0 also provided PCR positive results after 12 months, suggesting early detection by multiepitope proteins.

## Discussion

Serological methods are powerful tools in CVL diagnosis, being frequently used for canine mass screening. Several *Leishmania* antigens have been characterized, and recombinant technology has been used for the development of new enzymatic immunoassays [16], [28], [29]. Given the lack of sensitivity of Brazilian recommended assays to CVL diagnosis, mainly in asymptomatic dogs [8], [9], the search for new antigens is still needed.

In this work, we have produced and validated two new recombinant antigens for CVL diagnosis. They are multiepitope proteins, resulting from synthetic gene design, which has been referred elsewhere as a useful methodology [30], [31]. The validation of immunossorbent assays with our recombinant proteins was a very important contribution, because many antigens have not been tested in multicentric studies with a large panel of samples, mainly from uninfected animals, as we performed.

Other multiepitope proteins have already been used for the diagnosis of *Leishmania*-infected dogs, showing promising results [17], [18]. However, this is the first time that serological results are similar to those of parasitology, providing an early serological *Leishmania* diagnosis.

Our results indicate that the main usefulness of ELISA-PQs is in serological screening tests, once sensitivities exceeded 80%. However, specificities were about 66%, indicating a need for other confirmatory tests with better specificity. As stated elsewhere [32], the use of only one antigen in screening and confirmation tests in CVL diagnosis increases the number of false-positives. Furthermore, our multiepitope proteins could be useful in canine management due to their ability to detect infected animals with similar sensitivity to PCR, in a serological assay. Main advantages of using these multiepitope proteins are low cost and easy automation in ELISA tests.

ELISA-PQs showed moderate specificities with samples that had two concordant serological tests. This criterion of sample election is adopted by many authors [33]–[35]. However, it could have included infected dogs (before seroconversion) in the group of uninfected animals that were not detected by EIE-LVC kit and RIFI. On the other hand, our antigens are able to detect CVL infection before serological tests, as well as a molecular test (PCR); hence, the false-positive results could be correct (not false). Another explanation regarding low specificity could be the occurrence of cross reactions, leading to positive results in group N1 (dogs born in kennels with wire mesh in a non-endemic area dogs). Besides, when analyzing the false-negative results from ELISA-PQs, we observed that most of these samples (90.5%) presented titers lower than 1∶320, suggesting that our multiepitope proteins have difficulties in reacting with low titer samples. Similar to our results, Reis et al. [34] showed 12.5% of false-negative results when testing ELISA-k39, due to the presence of dogs with antibody titers lower than 1∶320. Other authors [14] have showed 15% of false-negative results also due to low antibody titers, in a latex agglutination test with the A2 antigen.

Detection of asymptomatic dogs in ELISA-PQs (80% of tested samples) was substantially superior compared to the detection by DPP or EIE-LVC kit. This is a relevant issue regarding visceral leishmaniasis control, as asymptomatic dogs play an important role in the epidemiological cycle of the disease [11]. Similar to our data, Martins et al. [16] also showed promising results with the recombinant protein LiHyp1, which detected 94% of asymptomatic dogs. In high transmission areas of visceral leishmaniasis, with prevalence above 3%, asymptomatic dogs must be included in control measures to guarantee program success [36]. Prevalence of CVL is very changeable in different areas in the world and also in different regions of the same country, as observed in Brazil. So, the use of a sorological test should consider this prevalence once it influences in negative and positive predictive values of a test. Although in low prevalence our proteins could not be the most useful antigens, they are still helpful tools to detect early infection. An important point relative to the detection of asymptomatic dogs is the ability to follow up on resistant animals. Thus, the described proteins could be used in the future to detect and follow up canine infection.

Our multiepitope proteins were able to detect circulating antibodies in the early phase of infection. All serum samples which were positive in ELISA-PQs were also positive in PCR thereafter. Positivities detected by ELISA-PQs, as well as by PCR, were not accompanied by EIE-LVC kit. As already stated, PCR provides positive results before serological tests [21], [37]. However, immunoassays with PQs apparently detect other types of immunoglobulins. One such case could be detection of IgMs, which are rapidly and transiently produced in the initial stage of CVL infection. This immunoglobulin could have been detected by the enzyme-conjugated antibody used in our ELISA-PQ tests (anti whole molecule). Some authors [38] have shown that canine IgG levels increase from the second month after experimental infection. Theoretically, from this point on, it is possible to serologically detect the infection, which occurred with ELISA-PQs. However, few studies are found in the literature about serological early detection of *L. infantum* infection. Quinnell et al. [21] showed that serology failed to detect 43 of 343 (12.5%) confirmed post-infection samples; 36 of these were from dogs early in infection (prior to seroconversion). In an experimental work [39], *Leishmania* infection was detected by ELISA with crude antigen in 37% of the dogs after 90 days. Similarly, Rodríguez-Cortés et al. [40] have detected 33.3% of dogs the same time post-infection.

Faria et al. [8] have described the peptides that compose PQ10 structure. When these synthetic peptides were tested together in ELISA, mixed in a single solution, the sensitivity was 75% and specificity, 95% in canine diagnosis. In comparison to multiepitope protein PQ10, an increase in sensitivity (88.8%) and also a decrease in specificity (80%) were observed. Thus, depending upon the use of the antigen, multiepitope protein or mixed peptides could be chosen, in order to have higher sensitivities or specificities.

Recent studies have evaluated the development of immunochromatographic tests with recombinant antigens to detect different pathologies [41]–[43], representing a possible approach for our proteins. These recombinant multiepitope antigens could be combined in an attempt to improve accuracy. Ideally, it would be interesting to use an antigen with good sensitivity, as observed with our multiepitope proteins, and another antigen with good specificity in the same strip test.

In conclusion, we have designed two new multiepitope recombinant proteins to improve CVL serodiagnosis. Our findings indicate that PQ10 and PQ20 could be useful for serodiagnosis and allow for the detection of asymptomatic dogs, as well as dogs in early phase of infection. The development of an immunochromatographic test using these proteins would be a valuable tool for CVL diagnosis.
